# Anticancer effects of OSW-1 on glioma cells via regulation of the PI3K/AKT signal pathway: A network pharmacology approach and experimental validation *in vitro* and *in vivo*

**DOI:** 10.3389/fphar.2022.967141

**Published:** 2022-09-05

**Authors:** Zhixin Zhan, Ziqiang Liu, Chaochao Zhang, Haijun Gao, Jiacheng Lai, Yong Chen, Haiyan Huang

**Affiliations:** Department of Neurosurgery, The First Hospital of Jilin University, Changchun, China

**Keywords:** OSW-1, glioma, network pharmacology, cell cycle, apoptosis, Pi3k/ AKt

## Abstract

**Background:** Gliomas are the most common primary intracranial malignant tumors with poor prognosis, despite the remarkable advances in medical technology that have been made. OSW-1, isolated from Ornithogalum saundersiae, possesses anticancer activity against various malignant cancer cells. However, the effects of OSW-1 on gliomas and its potential mechanisms remain unclear.

**Methods:** Network pharmacology was employed for predicting potential key targets and mechanisms of the anticancer effects of OSW-1 on glioma. Experiments, including the Cell Counting Kit-8, colony formation, and flow cytometry, were performed to investigate how OSW-1 affects the biological behavior of glioma cells *in vitro*. Western blotting was used to detect changes in related proteins, such as those involved in the cell cycle, apoptosis, and signaling pathways. The nude mouse xenograft model was used to detect the effect of OSW-1 on inhibiting the proliferation of glioma cells *in vivo*.

**Results:** An “OSW-1-Targets-Glioma” intersection network consisting of 151 intersecting genes was acquired to construct a “Protein–Protein Interaction network” and predict the top 10 core targets. According to the Kyoto Encyclopedia of Genes and Genomes pathway analysis, the PI3K/AKT signaling pathway was the top 3-ranked pathway, with 38 enriched intersecting genes. The glioma T98G and LN18 cell lines were used to verify the predictions. OSW-1 significantly inhibited the viability and proliferation of glioma cells in a dose- and time-dependent manner. Flow cytometry showed that OSW-1 arrested the cell cycle at the G2/M phase, and the apoptotic ratio of glioma cells increased significantly with increasing concentrations. Western blotting revealed that the expression levels of p-PI3K and p-AKT1 in glioma cells treated with OSW-1 were significantly lower than those in the controls; however, 740Y-P, a PI3K activator, significantly reversed the inactivation of the PI3K/AKT signaling pathway caused by OSW-1. Furthermore, the mouse xenograft model confirmed the suppressive effect of OSW-1 on tumor growth *in vivo*.

**Conclusion:** OSW-1 is a promising anti-glioma chemotherapeutic drug owing to its anticancer effects via downregulation of the PI3K/AKT signaling pathway. However, OSW-1 still has a long way to go to become a real anti-glioma drug.

## Introduction

Gliomas originating from the glial cells of the central nervous system (CNS) are the most common primary brain tumors. Except for pilocytic astrocytomas, gliomas are normally associated with poor prognosis and poor quality of life (Ohgaki and Kleihues, 2005). Currently, the main treatment for newly diagnosed gliomas is based on the classical Stupp protocol, including surgical resection combined with postoperative radiotherapy and chemotherapy using temozolomide (TMZ) (Stupp et al., 2005). Furthermore, immunotherapy, targeted therapy, and other novel therapeutic options have made great progress in the treatment of glioma. However, the overall outcome remains unsatisfactory (Omuro and DeAngelis, 2013). Especially for glioblastoma (GBM), the most common (57.3%) and malignant (grade IV) glioma type, the median survival time is 14–17 months and the 5-years survival rate is only 5% (Stupp et al., 2009; Reifenberger et al., 2017). Therefore, there is an urgent need to develop innovative therapeutic methods or discover effective anticancer agents to improve the prognosis of gliomas.

Natural compounds extracted from traditional medicinal herbs and plants have become valuable sources of clinically useful anticancer agents (Buyel, 2018). Compared to conventional chemotherapeutic drugs, natural compounds are more effective in inhibiting tumor growth with low toxicity and are less likely to cause drug tolerance for multiple targets (Kumar and Jaitak, 2019). Thus, natural compounds are promising anticancer agents. In Chinese folk medicine, Ornithogalum saundersiae is commonly used to treat hepatitis and some cancers owing to its anti-inflammatory and antitumor properties (Zhan et al., 2021). In 1992, Kubo et al. isolated a steroidal saponin, OSW-1, from O. saundersiae bulbs (Kubo et al., 1992). It was then shown to have higher cytotoxicity against malignant cancer cells than many clinical chemotherapeutic drugs such as doxorubicin, camptothecin, and paclitaxel (Mimaki et al., 1997). Interestingly, cancer cells are more sensitive to this cytotoxicity than normal cells, such that the IC_50_ of OSW-1 is 40–150-folds higher than that observed in malignant cells (Zhou et al., 2005). The potent anticancer effects have prompted many preclinical studies on its role in oncotherapy (Zhan et al., 2021). However, identifying the molecular targets of OSW-1 is challenging because of its complex structure. To date, the anticancer mechanisms of OSW-1 remain largely unclear, limiting its clinical applications.

**FIGURE 1:**
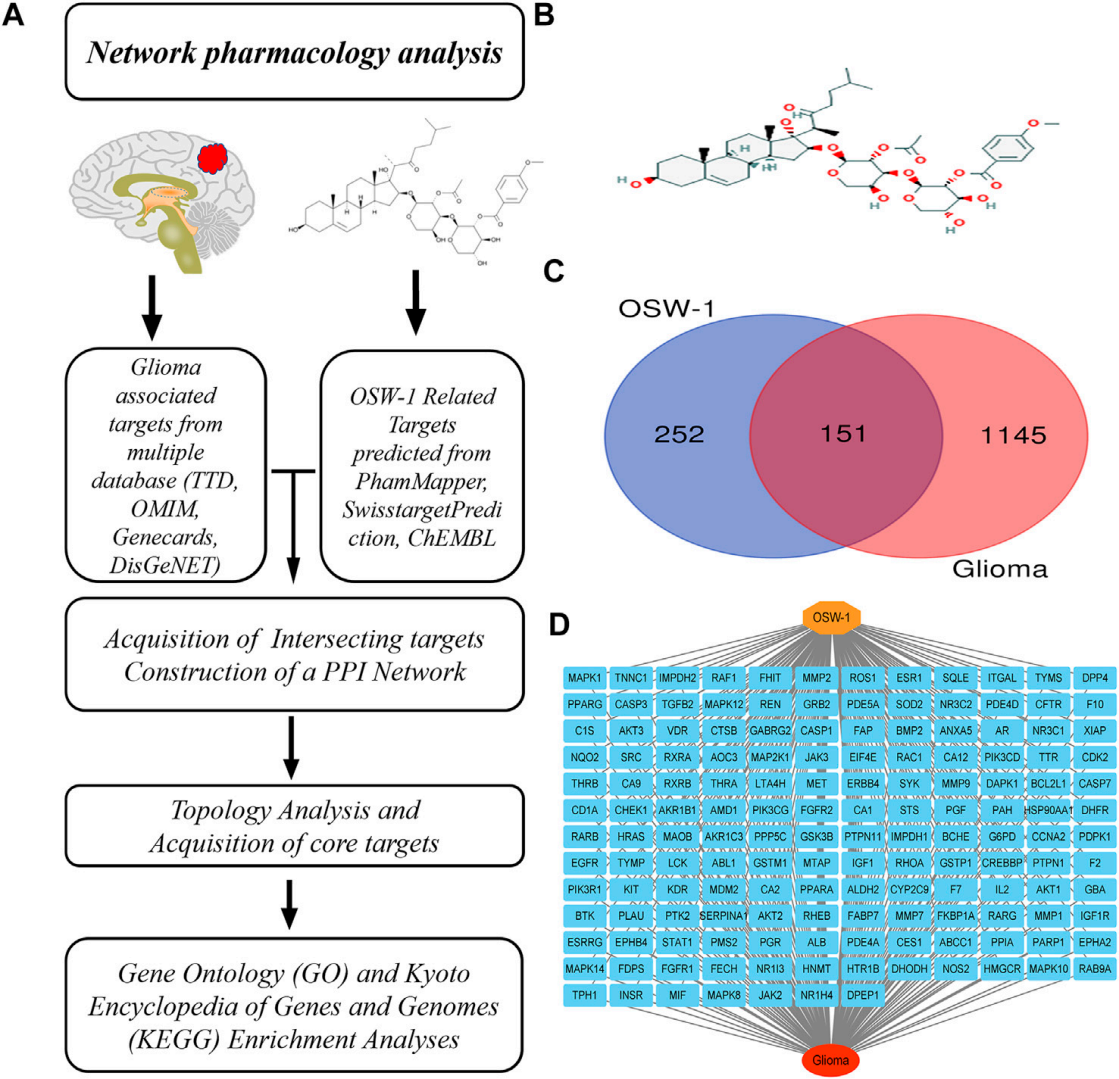
Network pharmacology of OSW-1 on glioma **(A)** The schematic flowchart of the present network pharmacology analysis **(B)** The 2D chemical structure of OSW-1 **(C)** The Venn map of OSW-1-targets and Glioma-related genes **(D)** The “OSW-1-Targets-Glioma” intersection network.

In this study, network pharmacology, a new paradigm in drug discovery, was employed to predict the potential key targets and mechanisms of OSW-1 (Hopkins, 2008) to reveal the possible signaling pathways involved in its anti-glioma effects (Figure 1A). In addition, biological experiments were performed to investigate how OSW-1 affects the survival and death of glioma cells and to verify the mechanisms underlying these phenomena. To the best of our knowledge, this is the first study to describe OSW-1 as an anticancer agent for glioma *in vitro* and *in vivo* and to uncover its mechanisms of regulating the PI3K/AKT signaling pathway.

## Materials and methods

### Chemicals and reagents

OSW-1 (C47H68O15, purity ≥95%) was purchased from Cayman Chemical Company (Ann Arbor, Michigan, United States), dissolved in dimethyl-sulfoxide DMSO (Sigma-Aldrich, St. Louis, MO, United States) then stored in the dark at − 20°C. OSW-1 was diluted with cell culture medium to the required concentrations, with DMSO concentration <0.1%, to avoid side effects. Dulbecco’s modified Eagle medium (DMEM) and fetal bovine serum (FBS) were purchased from Thermo Fisher Scientific (Gibco, United States). Cell counting Kit-8 (CCK-8), RNase, penicillin/streptomycin, propidium iodide, Annexin V-FITC Apoptosis Detection Kit, RIPA lysate, protease inhibitor, protein phosphatase inhibitor, BCA protein assay kit, and electrochemiluminescence (ECL) kit were purchased from Beyotime Biotechnology Company (Shanghai, China). 740Y-P was purchased from TargetMol (Shanghai, China). Primary antibodies against P21 (ab109520), cyclin B1 (ab32053), CDK1 (ab133327), PARP-1 (ab191217), cleaved-PARP-1 (ab32064), cleaved caspase-3 (ab214430), beta Actin (ab8226), PI3K(ab191606), p-PI3K (ab278545), AKT1 (ab183556), and p-AKT1 (ab81283) were purchased from Abcam (Cambridge, MA, United Kingdom). Primary antibodies against cleaved caspase-9 (#9502) and secondary antibodies (goat anti-mouse IgG and goat anti-rabbit IgG, #98164 and #91196) were purchased from Cell Signaling Technology (Beverly, MA, United States).

### Acquisition of OSW-1 potential targets and glioma-associated genes

PharmMapper Server (http://lilab-ecust.cn/pharmmapper/) is an updated integrated pharmacophore-matching platform with a statistical method for predicting compound-associated protein targets. In this study, the SDF file containing the chemical structure of OSW-1 was downloaded from the PubChem database (https://pubchem. ncbi. nlm.nih.gov/) and uploaded to PharmMapper to obtain potential targets. ChEMBL (https://www.ebi.ac.uk/chembl/) (Mendez et al., 2019) and SwisstargetPrediction (http://www.swisstargetpredictionch/) (Daina et al., 2019) are also used to screen the targets. The predicted results were integrated. In addition, we acquired glioma-associated gene targets, by using “glioma” as a keyword, from the GeneCard database (https://www.GeneCards.org/) (Safran et al., 2010), Therapeutic Target Database (http://bidd.nus.edu.sg/group/cjttd/) (Santos et al., 2017), DisGeNET database (https://www.disgenet.org/) (Piñero et al., 2017), and OMIM database (http://www.omim.org/) (Amberger et al., 2015). Then we integrated the results and obtained potential glioma-associated targets. UniProt (https://www.uniprot.org/) was used to improve the UniProt ID and gene name of the target.

### Acquisition of intersecting genes and Protein–Protein Interaction (PPI) network construction and analysis

Potential targets and glioma-associated genes were uploaded to the Jvenn website (http://jvenn.toulouse.inra.fr/app/example.html) to determine the intersecting genes, which were considered candidate targets of OSW-1 for treating glioma. An “OSW-1-Targets-Glioma” network was constructed by importing these genes into the Cytoscape software (version 3.8.2). To investigate the relationship between these targets, intersecting genes were imported into the STRING database (https://string-db.org/) to construct a PPI network that was subsequently visualized using Cytoscape software. Using CytoHubba in Cytoscape, we ranked the nodes in the network according to the degree algorithm and obtained the top 10 core genes.

### Gene ontology (GO) and Kyoto Encyclopedia of Genes and Genomes (KEGG) enrichment analyses

DAVID is a bioinformatics database that integrates biological data and analysis tools to provide researchers with a comprehensive set of functional annotation tools to understand the biological meaning behind a large number of genes. We imported the intersecting genes into the Database for Annotation, Visualization, and Integrated Discovery (DAVID) (version 6.8) (https://david.ncifcrf.gov/home.jsp) for GO enrichment analysis, which evaluated the participation of the targets in terms of their biological process (BP), cell component (CC), and molecular function (MF). In addition, we carried out KEGG enrichment analysis for the systematic analysis of signaling pathways in which these genes may be involved within cells. The GO and KEGG results were imported into the WeiShengxin website (http://www.bioinformatics.com.cn/en) for annotation and visualization.

### Cell culture

Human glioma T98G and LN18 cells were purchased from the American Type Culture Collection (ATCC; Manassas, VA, United States) and were preserved in our laboratory at the Translational Medicine Institute of the First Hospital of Jilin University. T98G cells were cultured in DMEM supplemented with 10% FBS and 1% penicillin/streptomycin in 5% CO_2_ atmosphere at 37°C.

### Cell viability assay

A Cell Counting Kit-8 (CCK-8) was used to analyze the inhibitory effect of OSW-1 on the viability of glioma T98G and LN18 cells. T98G and LN18 cells (5× 10^3^ cells/well) were seeded into 96-well plates and incubated overnight. The following day, the original cell culture medium was replaced with fresh medium containing different concentrations (0, 0.01, 0.1, 1, 10, 100 nM) of OSW-1 for 24, 48, and 72 h. Then, 10 μL of CCK-8 solution was added to each well, and cells were incubated at 37°C for 1 h. The optical density (OD) of each well was measured at 460 nm using a microplate reader (BioTek Instruments, United States).

### Clone formation experiment

T98G and LN18 cells were seeded into a 6-well plate at a density of 1 × 10^3^ cells/well and cultured overnight to enable attachment to the walls. The following day, media containing OSW-1 at different concentrations (0, 0.1, 1, 10 nM) were added to each well for cells cultured for 24 h. After 24 h, the medium was removed, and fresh medium was added to replace the old medium of each well every 3 days for approximately 10 days. When tumor cells formed a colony of approximately 50 cells, contents were carefully washed three times with PBS, fixed with 4% paraformaldehyde for 30 min, stained with 0.1% crystal violet solution for 20 min, and finally photographed.

### Cell-cycle assay

T98G and LN18 cells were seeded into 6-well plates at a density of 3 × 10^5^ cells/well and cultured overnight. The cells were incubated with different concentrations of OSW-1 (0, 0.1, 1, 10 nM) for 24 h. After that, the cells were collected and fixed in 70% ethanol at −20°C for 24 h. The following day, cells were stained with PI and RNase in the dark for 20 min. The cell cycle distribution was detected using flow cytometry (Becton Dickinson, San Diego, CA, United States) and analyzed using the FlowJo 10.3 software (TreeStar, United States).

### Apoptotic assay

The Annexin V-FITC/PI apoptosis detection kit was used to evaluate the apoptosis of glioma T98G and LN18cells induced by OSW-1 according to the manufacturer’s protocol. T98G and LN18 cells were seeded in six-well plates at a density of 5×10^5^ cells/well. After the cells attached to the wall, they were incubated with different concentrations of OSW-1 (0, 0.1, 1, 10 nM) for 24 h. Then, the cells were collected and incubated with 500 μL 1ⅹAnnexin V Bing Buffer with 5 μL Annexin V-FITC and 5 μL PI for 20 min. We determined the apoptosis rate of cells using flow cytometry, and the results were analyzed using the FlowJo software.

### Western blotting

The RIPA buffer containing the phosphatase and protease inhibitors was employed to lyse tumor tissues and cultured glioma cells on ice to extract proteins. A BCA kit was used to determine the protein concentration. An equal amount of protein samples (containing 20 μg of total protein) were separated on an SDS-PAGE gel (8–12.5%) and transferred to a polyvinylidene fluoride (PVDF) membrane. The membranes were blocked in TBS with 5% non-fat dried milk at room temperature for 1 h and then left to incubate with the corresponding primary antibody at 4°C overnight. After the membranes were washed thrice with TBS-T for 10 min, they were incubated with the corresponding secondary antibody at room temperature for 1 h. Finally, the membranes were washed thrice with TBST for 10 min, and the protein bands were detected using ECL luminescence. The densitometry of the bands was analyzed using the ImageJ software (v1.52°, NIH, Bethesda, MD, United States).

### Xenograft tumor model

Animal experiments were approved by the Ethics Committee of the First Hospital of Jilin University (Changchun, China) and followed the five freedom and 3R principle (Chinese Association for Laboratory Animal Sciences). BALB/c-nu male mice (5–6 weeks of age, weighing 15–17 g) were purchased from the Charles River Experimental Animal Center (Beijing, China). Mice were housed in a specific pathogen-free environment at a temperature of 24 ± 2°C and humidity of 45 ± 10% on a 12 h:12 h light/dark cycle with food and water available *ad libitum*. Under sterile conditions, a 200-μL suspension (100 μL PBS and 100 μL Matrigel) of LN18 cells (1× 10^7^ cells/mouse) was inoculated subcutaneously into the right armpit of nude mice. When the tumor volume reached 200 mm^3^, the mice were randomly divided into two groups with five mice in each group. The mice from the experimental and control groups were intraperitoneally injected with OSW-1 (0.01 mg/kg, diluted in 100 μL PBS) and the same volume of saline, respectively, every day for consecutive 21 days. The long diameter (L) and short diameter (S) of the tumor were measured once every 3 days. Tumor volume (Tv) was calculated by the following formula: Tv = 0.5 × L × S^2^. The weight of each mouse was measured every 2 days. On the 21st day after administration, all mice were sacrificed. The tumor tissue was harvested and photographed, and the weight of the tumors was measured. Three tumor tissues were randomly selected from each group. After fully grinding the tumor tissues on ice, 10 µL RIPA buffer was added to each milligram of tumor tissue to extract tissue proteins. Then Western blotting was performed as described above.

### Statistical analyses

All experiments were repeated independently at least three times, and the experimental data are expressed as mean ± standard deviation. Statistical analyses were performed using one-way analysis of variance (ANOVA). *p*-values indicated in each figure, including *p < 0.05, **p < 0.01, ***p < 0.001, indicate significance. All statistical data were analyzed using the GraphPad Prism software (version 8.0; GraphPad Software Inc., La Jolla, CA, United States).

## Results

### Screening and identification of the potential targets of OSW-1 for treating glioma

The 2D chemical structure of OSW-1 was obtained from the PubChem database (https://pubchem.ncbi.nlm.nih.gov/compound/9854230#section=2D-Structure) and is shown in Figure 1B. After uploading the SDF file of OSW-1 to the PharmMapper database (default setting), 383 potential binding targets were identified. A total of 6 and 30 pharmacological targets were predicted by the SwissTargetPrediction online database (Probability >0) and ChEMBL (confidence = 90%, active, *Homo sapiens*), respectively. When we delete three duplicate and false items, we finally get 403 potentail targets of OSW-1. Furthermore, 626 glioma-associated targets were obtained from the Therapeutic Target Database, 31 targets were obtained from the OMIM database, 735 targets were obtained from the GeneCards database (Relevance score ≥2), and 16 targets were obtained from the DisGeNET database (Score ≥0.5). Based on the integrated results of the above databases, 1296 glioma-associated targets were predicted after deleting duplicate and false-positive items. In addition, a Venn diagram (Figure 1C) was generated by uploading the potential targets and glioma-associated genes to the Jvenn website. As shown in “OSW-1-Targets-Glioma” intersection network (Figure 1D), these 151 intersecting genes may be the key targets for the anticancer effects of OSW-1 on glioma.

### PPI network analysis and prediction of core targets

**FIGURE 2:**
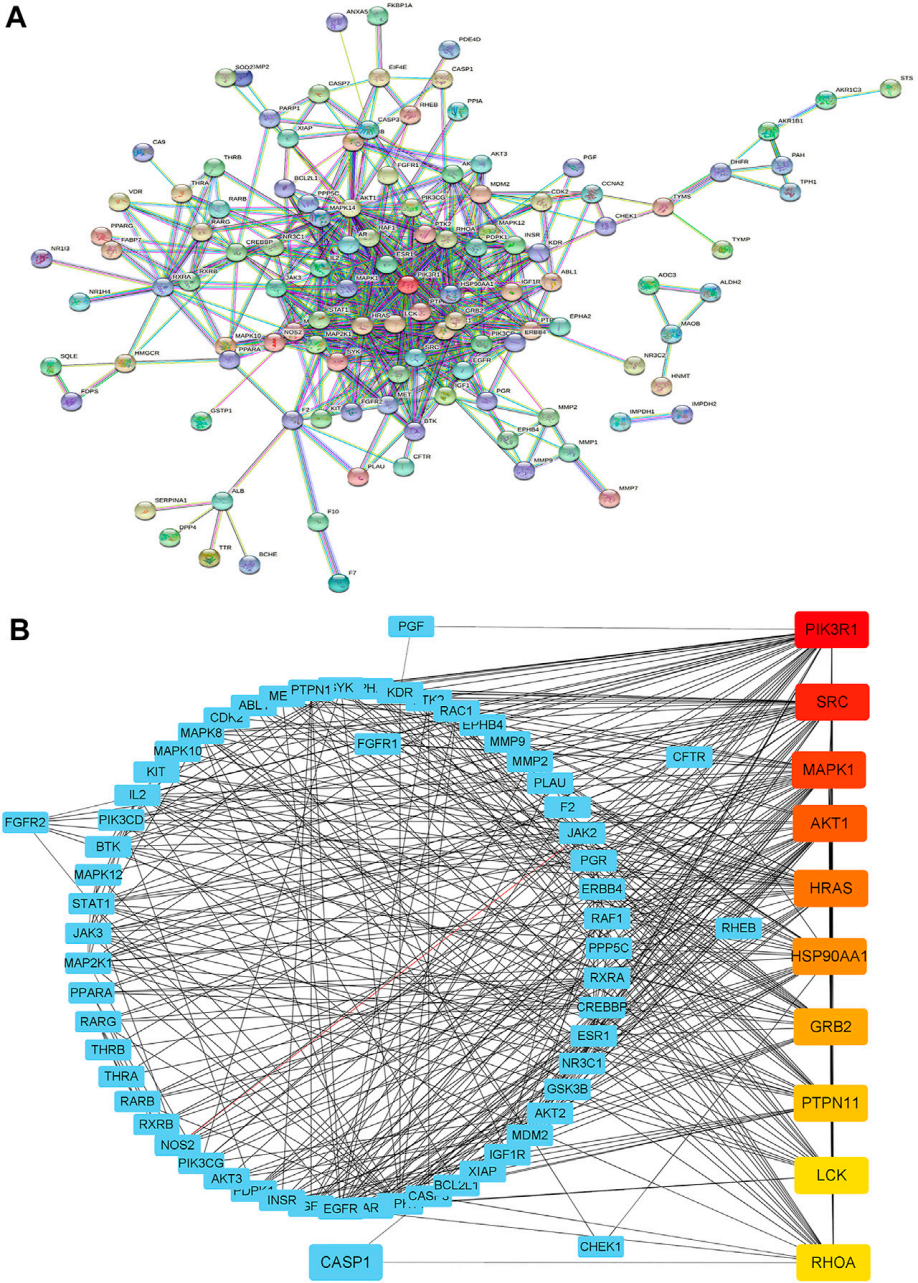
Construction of a PPI network **(A)** Constructing the PPI network by uploading intersecting genes to the STRING database **(B)** Ranking the top 10 hub genes of the PPI network using CytoHubba in Cytoscape. The darker the red, the higher the degree of connection.

To further investigate the relationship between these targets, a PPI network with 151 nodes and 518 edges (Figure 2A) was constructed by uploading these intersecting genes to the STRING database (minimum required interaction score = 0.9). Then, we sent the PPI network into the Cytoscape software for topological analysis and visualization. Using CytoHubba in Cytoscape, 10 hub genes (ranked in the order of degrees of freedom from high to low) with potentially crucial pathway roles were obtained: PI3KR1, SRC, MAPK1, AKT1, HRAS, HSP90AA1, GRB2, PEPN11, LCK, and RHOA (Figure 2B). PI3KR1 is the top one node with 42 degree of connection, which means that it may play an important role in the anti-glioma mechanism of OSW-1.

### GO and KEGG pathway enrichment analyses

**FIGURE 3:**
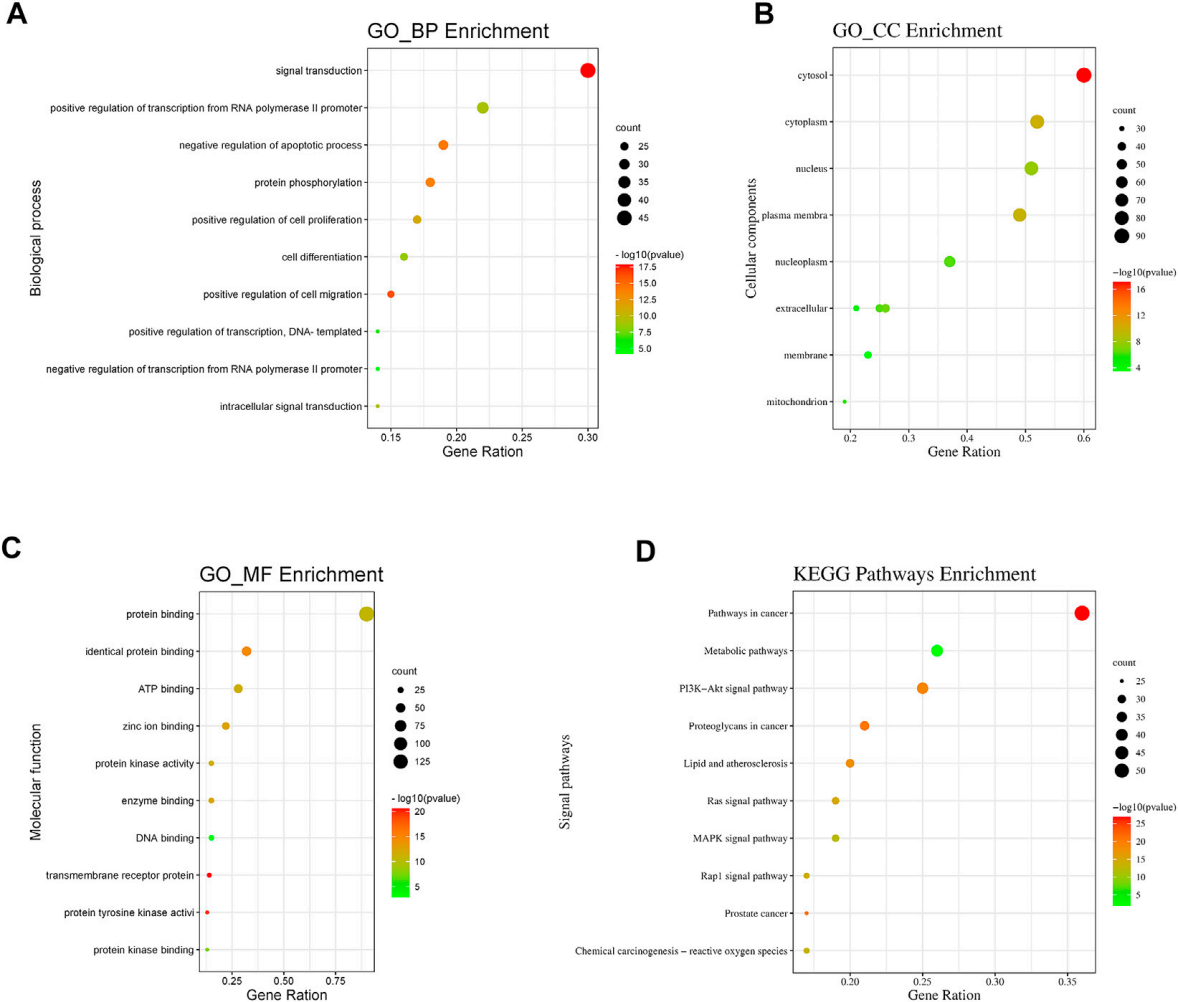
GO and KEGG pathway analysis of intersecting genes **(A,B,C)** Bubble plot for top 10 biological processes, cellular components and molecular functions of the GO enrichment analysis **(D)** Bubble plot for top 10 signaling pathways of the KEGG pathway enrichment analysis.

GO annotation and KEGG pathway analysis of the 151 intersecting genes of OSW-1 acting on glioma were performed using DAVID, and the results were visualized using the WeiShengxin website. BP, CC, and MF terms were used to describe GO function annotation analysis. We obtained 435 GO entries, including 386 BPs, 51 CCs, and 98 MFs that had *p*-values < 0.05, and the top 10 results were made into bubble charts (Figures 3A–C). As shown in Figure 3A, BP enrichment analysis revealed that OSW-1 may be involved in the regulation of signal transduction, apoptosis, cell proliferation, protein autophosphorylation, and cell migration. Furthermore, CC enrichment analysis showed that OSW-1 mainly plays a role in the cytosol, nucleus, and cytoplasm (Figure 3B). MF enrichment analysis suggested that the antitumor effects of OSW-1 might be related to its influence on protein, ATP, Zine ion, and enzyme binding and kinase activity. (Figure 3C). Additionally, we screened 146 pathways (*p*-values < 0.05) according to the results of KEGG analysis among these intersecting genes and found that the pathways in cancer, metabolic pathways, and PI3K/AKT signaling pathways were the top three highest-ranked significant pathways, with 54, 39, and 38 intersecting genes, respectively (Figure 3D). Intersecting genes in the PI3K/AKT signaling pathway play a critical role in the regulation of biological behavior in cancer cells, such as Raf-1, MEK, and ERK, which are involved in cell proliferation, angiogenesis, and DNA repair, whereas GSK3 and CDK are associated with the cell cycle. Additionally, the PI3K-AKT signaling pathway can affect the survival and apoptosis of cells through BCL2L1 and MDM2. These results suggested that the PI3K/AKT signal pathway may potentially play a role in the anti-glioma effects of OSW-1 by influencing cell proliferation, cell cycle progression, and apoptosis. The experiments, as described below, verified the hypothesis.

### OSW-1 inhibited cell viability and proliferation of glioma cells

**FIGURE 4:**
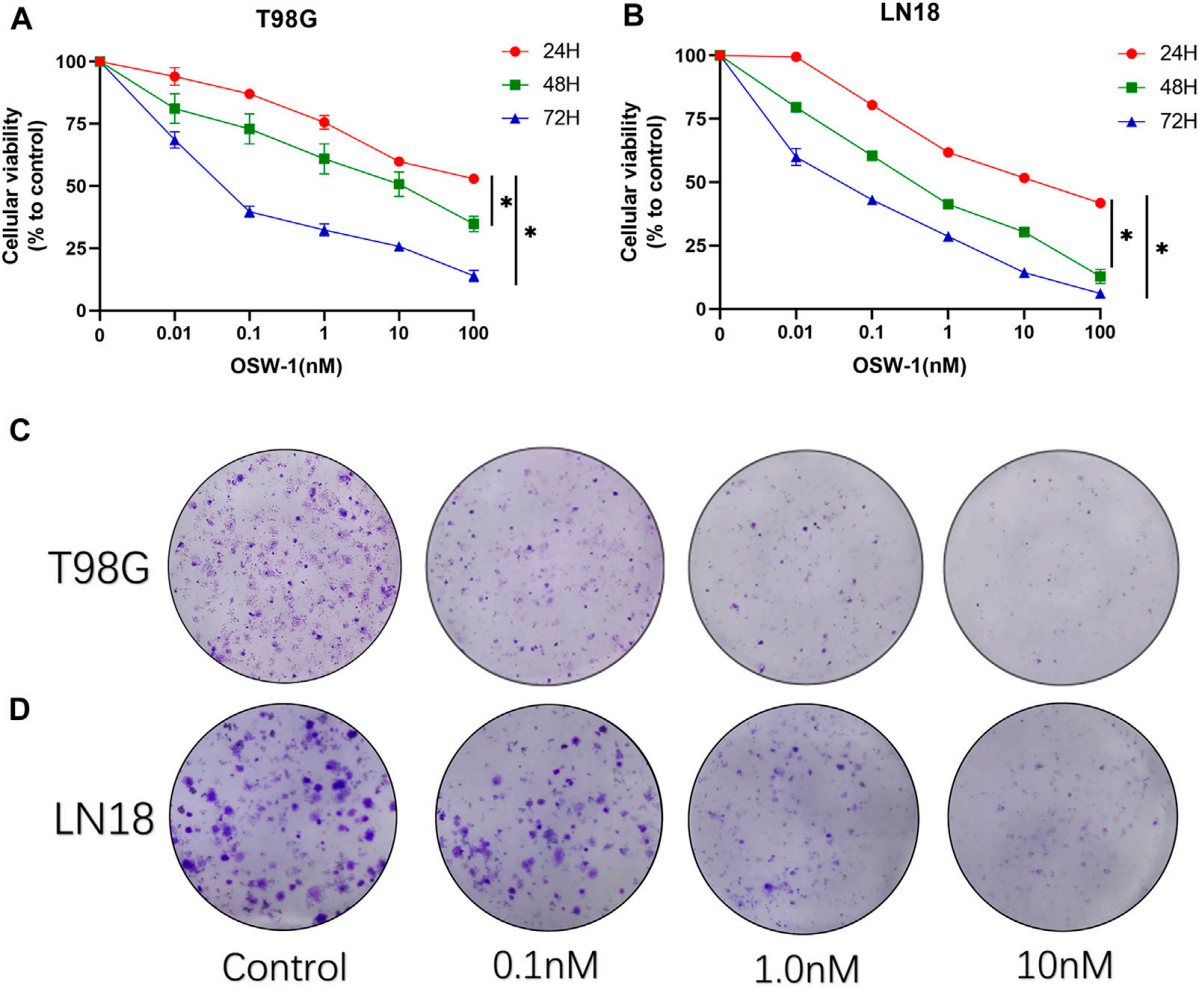
OSW-1 inhibited cell viability and proliferation of glioma cells **(A,B)** CCK-8 assay detected the viability of cells treated with different concentrations of OSW-1 for 24, 48, and 72 h. The results showed that OSW-1 inhibited cellular viability in a time- and dose-dependent manner **(C,D)** Clone formation experiments showed that clone numbers of glioma cells decreased gradually with increasing concentrations of OSW-1. *p < 0.05, compared to the control group.

We performed a CCK-8 assay to detect the inhibitory effects of OSW-1 on the viability and proliferation of glioma cells. The cells were treated with various concentrations of OSW-1 (0, 0.01, 0.1, 1, 10, and 100 nM) for 24, 48, and 72 h. The results showed that OSW-1 significantly reduced the viability of glioma cells in a dose- and time-dependent manner (Figures 4A,B). The 50% growth-inhibitory concentration (IC50) values of OSW-1 for T98G cells at 24, 48, and 72 h were 43.35, 13.02, and 0.07 nM, respectively. The IC50 values of OSW-1 for LN18 cells at 24, 48, and 72 h were 15.73, 0.45, and 0.04 nM, respectively. In addition, clone formation experiments were performed to evaluate the long-term effects of OSW-1 on the proliferation of individual glioma cells. The results suggested that the number of clones gradually decreased with increasing OSW-1 concentration, which indicated a decline in the proliferation ability of tumor cells (Figures 4C,D).

### OSW-1 arrested cell cycle at the G2/M phase in glioma cells

**FIGURE 5:**
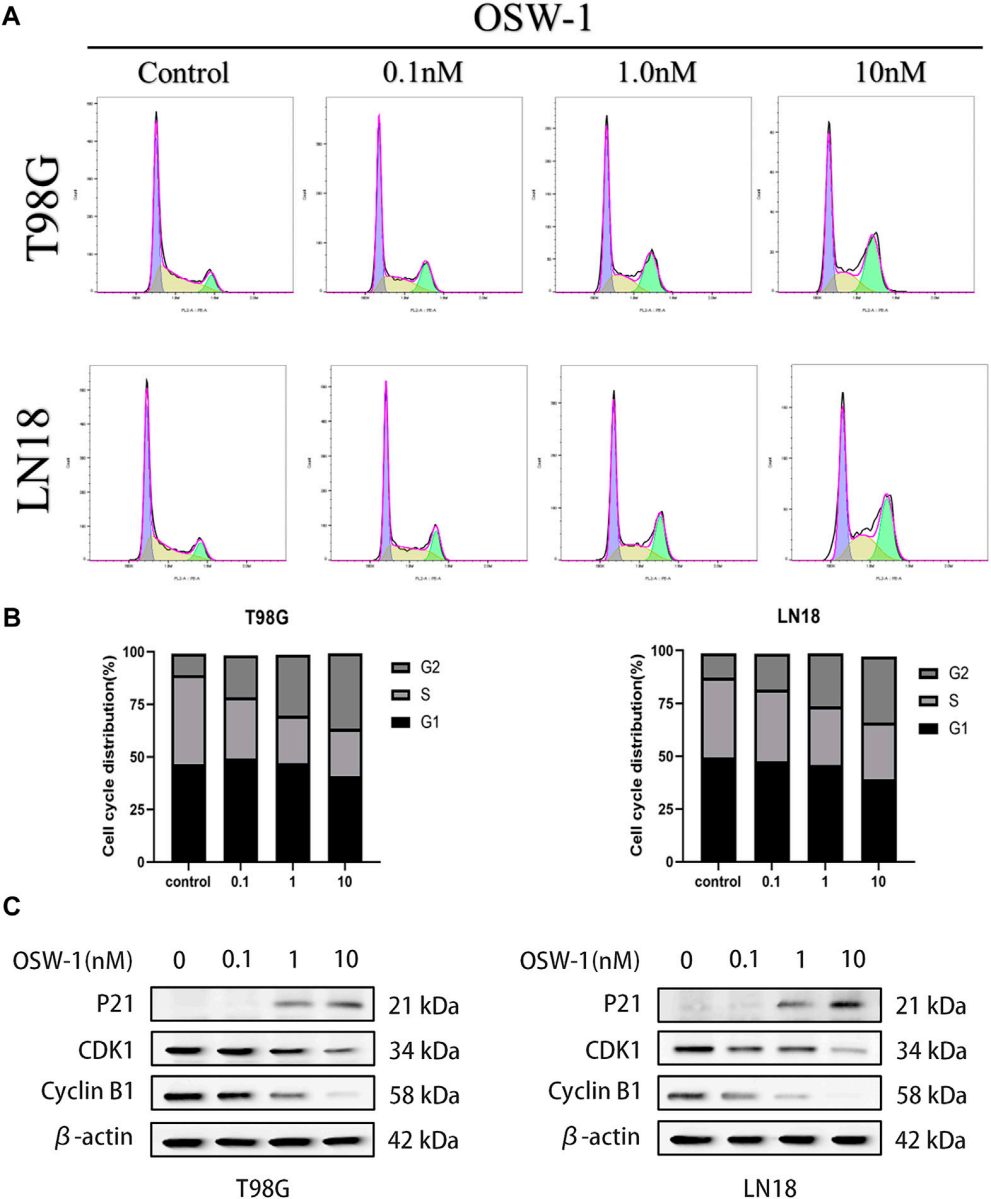
OSW-1 arrested cell cycle at the G2/M phase in glioma cells **(A)** Cell cycle changes of glioma cells treated with different concentrations of OSW-1 for 24 h were detected and analyzed using flow cytometry and the FlowJo software **(B)** Percentages of glioma cells in the G2/M phase were significantly increased in a dose-dependent manner **(C)** Western blot shows the changes of cell cycle-related proteins after OSW-1 treatment for 24 h.

Flow cytometry was used to detect cell cycle changes after treatment of glioma cells with OSW-1 for 24 h, and the FlowJo software was used to analyze the cell cycle distribution (Figure 5A). Compared with that of the control group, the proportion of glioma cells at the G2/M phase was significantly increased in the 10 nM OSW-1 group (Figure 5B). Furthermore, we detected the expression levels of cell cycle-related proteins using western blotting to explore the underlying mechanism. Western blotting results revealed that increasing the concentrations of OSW-1 led to the upregulation of p21 and downregulation of cyclin B1 and CDK1 (Figure 5C). These results demonstrate that OSW-1 arrested the cell cycle at the G2/M phase by regulating cell cycle-related protein levels in glioma cells.

### OSW-1 induced apoptosis in glioma cells

**FIGURE 6:**
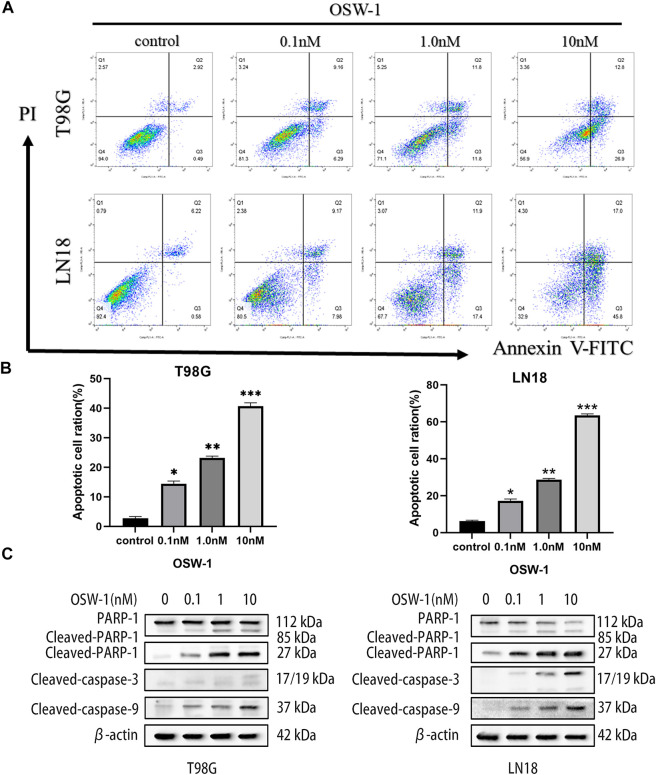
OSW-1 induced apoptosis in glioma cells **(A)** Flow cytometry was used to detected apoptosis of glioma cells treated with different concentrations of OSW-1 for 24 h, and results were analyzed using the FlowJo software **(B)** OSW-1 significantly increased the apoptotic cell ratio (Q2+Q3) of T98G and LN18 cells in a dose-dependent manner **(C)** Western blot shows the changes of apoptosis-related proteins after OSW-1 treatment for 24 h *p < 0.05, **p < 0.01 and ***p < 0.001, compared to the control group.

T98G and LN18 cells were stained with Annexin V-FITC/PI double staining solution and analyzed using flow cytometry to evaluate the effect of different concentrations of OSW-1 on apoptosis (Figure 6A). The results showed that OSW-1 treatment significantly increased the ratio of total apoptotic cells, including early and late apoptosis (Q3 + Q2) (Figure 6B). To further confirm these results, we detected apoptosis-related proteins using western blotting. Compared with the control group, OSW-1 significantly increased the expression of cleaved PARP-1 and cleaved Caspase-3/9 proteins but decreased the expression of PARP-1 protein in a dose-dependent manner (Figure 6C).

### OSW-1 inhibited the activation of the PI3K/AKT signaling pathway in glioma cells

**FIGURE 7:**
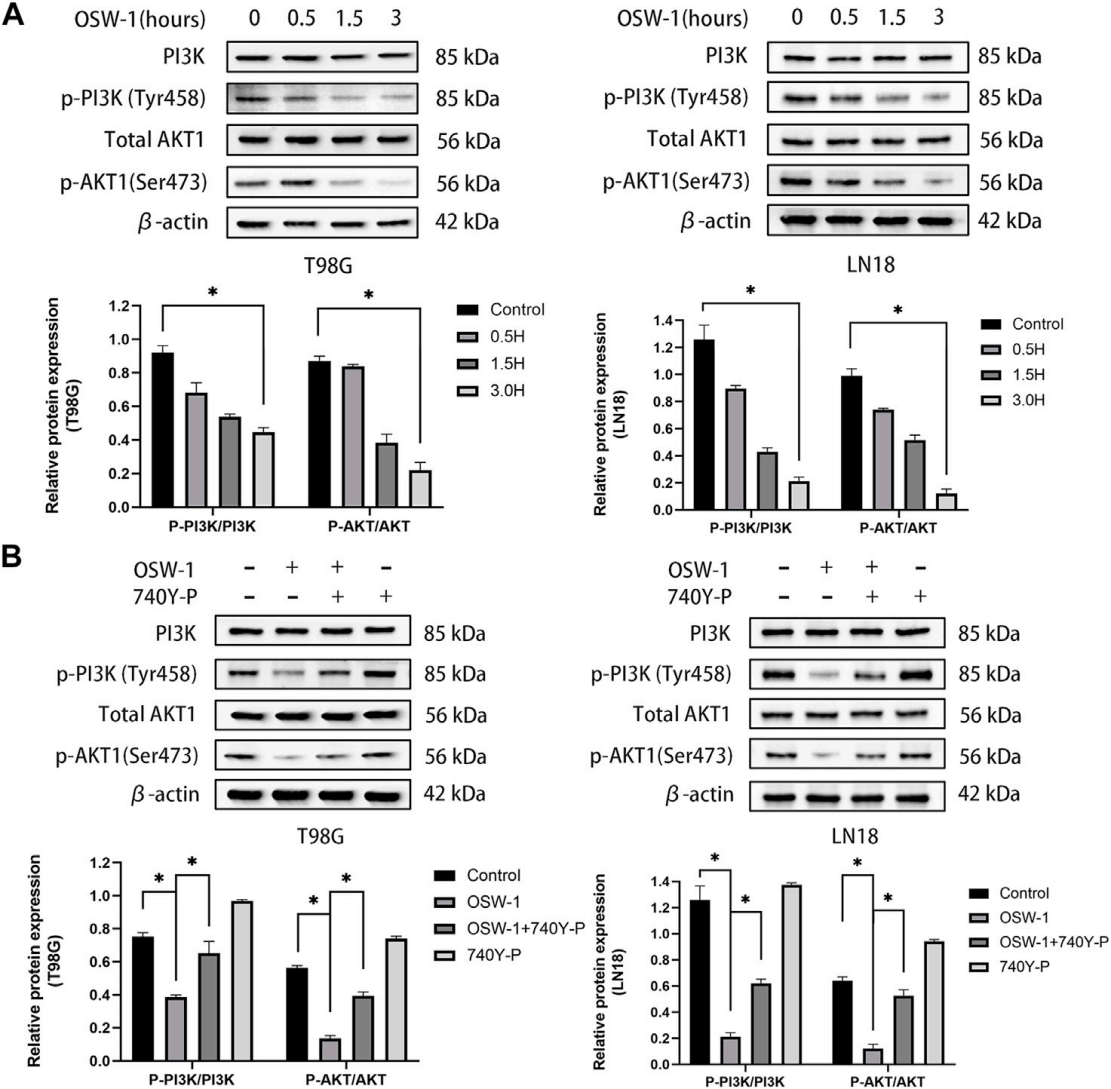
The inhibitory effects of OSW-1 on the PI3K/AKT signaling pathway in glioma cells can be reversed by 740Y-P **(A)** Western blot of AKT1, p-AKT1, PI3K, and p-PI3K in glioma cells treated with OSW-1 (10 nM) for different hours. OSW-1 significantly reduced the expression levels of p-Akt and p-PI3K at 3 h **(B)** Western blot of AKT1, p-AKT1, PI3K, and p-PI3K in glioma cells treated with OSW-1 (10 nM), 740Y-P (15 µM), OSW-1 (10 nM)+740Y-P (15 µM) or controls for 3 h. The expression levels of p-Akt and p-PI3K were significantly higher in the OSW-1 + 740Y-P-treated group compared with the OSW-1-treated group. *p < 0.05, compared to the control group.

The above experimental results demonstrated that OSW-1 can significantly inhibit cell growth, arrest the cell cycle, and induce apoptosis. The network pharmacology approach suggested that the PI3K/AKT signaling pathway may play a potential role in these anticancer effects. To test this hypothesis, we examined the expression levels of PI3K, p-PI3K, AKT1, and p-AKT1 in T98G and LN18 cells after 0.5, 1.5, and 3 h of treatment using OSW-1 (10 nM). Both p-PI3K and p-AKT1 levels were significantly lower in the group treated with OSW-1 for 3 h than in the control group (Figure 7A). These results suggested that OSW-1 inhibits the activation of PI3K and AKT1, indicating that the PI3K/AKT pathway is inhibited by OSW-1 in glioma cells.

### 740 Y-P reversed inactivation of the PI3K/AKT signaling pathway caused by OSW-1

To further clarify the role of OSW-1 in inhibiting the activation of the PI3K/AKT signaling pathway in glioma cells, we used the PI3K activator 740Y-P. T98G and LN18 cells were treated with OSW-1 (10 nM) alone, 740Y-P (15 µM) alone, or in combination with OSW-1 (10 nM) for 3 h. The same western blot assay was performed to detect the protein levels of p-PI3K, PI3K, p-AKT1, and AKT1. The expression levels of p-PI3K and p-Akt1 in glioma cells treated with OSW-1 alone were significantly lower than those in the controls; however, OSW-1 combined with 740Y-P significantly increased these levels (Figure 7B). These results suggest that 740Y-P can reverse the inhibitory effect of OSW-1 on the PI3K/AKT signaling pathway.

### OSW-1 inhibited glioma cell proliferation *in vivo*

**FIGURE 8:**
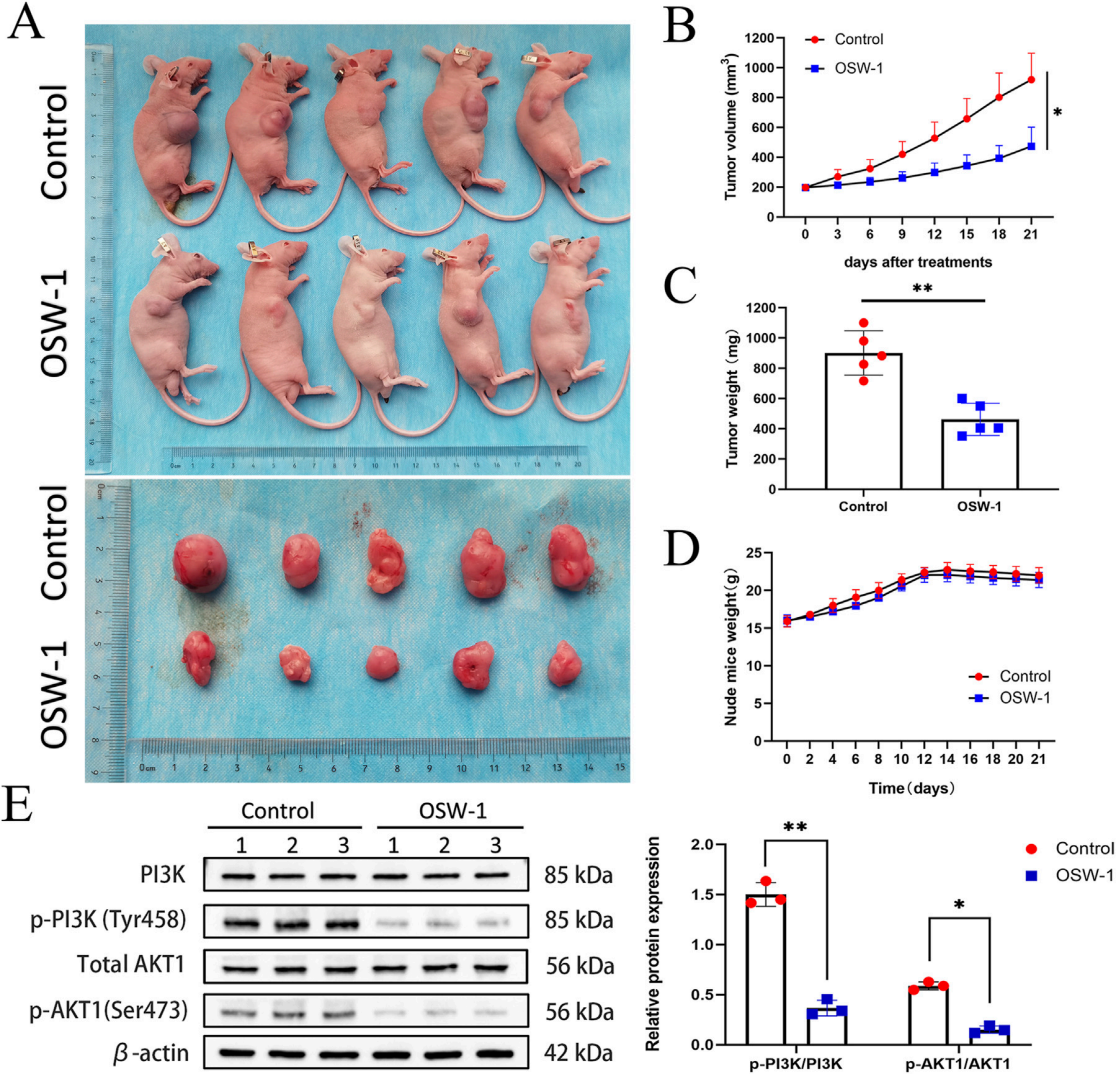
OSW-1 inhibited tumor growth in the xenograft model mice **(A)** The tumors of control mice and OSW-1-treated mice were extracted and photographed **(B,C)** Inhibitions in the average volume and weight of glioma xenografts were observed after 21 days of OSW-1 treatment **(D)** Body weight changes of mice during 21 days of exposure **(E)** Western blotting of tumor tissue lysates. *p < 0.05, **p < 0.01, compared to the control group.

All the nude mice grew well throughout the experimental process; none of the mice demonstrated evidence of infection, weight loss, adverse efects, or death. Gradual increase in the tumor volume of the nude mice was observed as the experiment progressed. The tumors in the control group were evidently larger compared to the OSW-1 treatment group (Figure 8A); particularly, an evident reductionin the average tumor volume and weight was observed in the OSW-1 group (Figures 8B,C). However, no signifcant diference was observed regarding the body weight between the two groups, indicating the low toxicity of OSW-1 *in vivo* (Figure 8D). The western blotting assays of tumor tissue lysates showed that OSW-1 inhibited the expression levels of p-PI3K and p-Akt1 in the OSW-1 treatment group compared to the control group (Figure 8E). Collectively, these results demonstrated OSW-1 inhibited glioma tumor progression by targeting PI3K/AkT signaling pathways *in vivo*.

## Discussion

OSW-1, a steroidal saponin, was first isolated from O. saundersiae bulbs by Kubo et al., in 1992 (Kubo et al., 1992). Many preclinical studies have confirmed its exceptional inhibitory activities against different cancer cells. However, the unclear anticancer mechanisms and low yield rate of OSW-1 limit its further clinical applications. For the past 30 years, the synthesis and structure–activity relationship of OSW-1 has been the major research focus. Recently, studies regarding the mechanism of OSW-1 have increased with the improved synthetic technology of OSW-1 and its derivatives. Zhan et al. summarized the available evidence on the anticancer effects and mechanisms of OSW-1 and found that OSW-1 exerts anticancer effects through various pathways and targets (Zhan et al., 2021). In prostate cancer, it can selectively obstruct the mTORC2 complex to inhibit cell growth (Elgehama, 2021). In ovarian cancer, it can target ORP4, which is associated with proliferative activity (Bensen et al., 2021); In breast cancer, it can decrease the expression of NFATc2 to inhibit tumor growth and metastasis (Ding et al., 2020). In cervical cancer, it can induce mislocalization of Oxysterol-Binding Protein and trigger the apoptotic Golgi stress response (Kimura et al., 2019). To date, there have been no studies on the anticancer mechanisms of OSW-1 in glioma, although OSW-1 has been reported to exhibit extremely potent cytotoxic activity against glioma U87-MG cells, with an IC50 value in the nanomolar concentration range (0.047 nM) (Zhou et al., 2005), indicating that it is a promising anti-glioma agent.

Gliomas represent approximately 26% of all brain and CNS tumors and are the most common primary intracranial malignant tumors, accounting for 82.4% (Ostrom et al., 2019). Depending on the type of glial cells from which the tumor originates, gliomas can be classified into multiple specific histological subtypes, such as ependymomas, astrocytomas (including GBM), oligodendrogliomas, and oligoastrocytomas. Although not all types consistently behave in a malignant fashion, gliomas are usually associated with poor prognosis, especially GBM and high-grade gliomas, which have a very short survival, high recurrence rate, and mortality (Omuro and DeAngelis, 2013; Ostrom et al., 2015). Gliomas are characterized by extensive invasive growth, making total surgical resection difficult. Currently, postoperative chemotherapy with temozolomide (TMZ) is frequently combined with radiotherapy as part of the first-line treatment for gliomas. However, the overall outcomes remain unsatisfactory, partially due to radio- and TMZ resistance (Ortiz et al., 2021). It has been reported that at least 50% of TMZ-treated patients do not respond to TMZ, and multiple GBM cell lines, such as LN-18, T98G, and U138, are naturally resistant to TMZ (Lee, 2016). For patients with TMZ resistance, a new anticancer drug that can replace TMZ is urgently needed. In this regard, we chose the T98G and LN18 cell lines for the *in vitro* experiments with OSW-1.

With the development of extraction and purification technologies, natural compounds that possess inhibitory effects against malignant cancer cells are attracting widespread attention and are considered promising anticancer therapeutic agents (Sauter, 2020). However, identifying the molecular targets of these natural anticancer agents is challenging (Kubczak et al., 2021). Network pharmacology, a new approach in drug discovery, is expected to deepen our understanding of drug action across multiple layers of information by establishing a “compound-protein/gene-disease” network and can reveal the regulation principles of small molecules in a more effective manner (Boezio et al., 2017; Zhang et al., 2019). OSW-1, a natural compound, has not been studied in gliomas, and the underlying mechanisms are not fully understood. Therefore, we employed network pharmacology to predict the potential molecular targets and signaling pathways involved. To the best of our knowledge, there have been no structure-function analyses based on the network pharmacology of OSW-1. In the present study, bioinformatic analysis databases and software were used to explore the potential targets of OSW-1. One hundred and fifty-one intersecting genes were obtained from the “OSW-1-Targets-Glioma” intersection network and subsequently, 10 hub geneswith potentially crucial pathway roles were identified (Figure 2B). These genes are associated with regulation of signal transduction, apoptosis, cell proliferation, protein autophosphorylation, and cell migration. According to the KEGG analysis, PI3K/AKT pathway was one of the highest-ranked significant pathways, with 38 enriched intersecting genes. Therefore, We speculated that OSW-1 may influence cell proliferation, apoptosis, and cell cycle progression in glioma by binding to some core proteins in the PI3K/AKT signaling pathway.

Cancer cells are characterized by unlimited proliferation and growth and can enter the cell cycle incessantly (Hanahan and Weinberg, 2011). Although the precise mechanism controlling the proliferative signals of cancer cells is unclear, cell cycle arrest is considered an effective way to inhibit uncontrolled cell proliferation (Suski et al., 2021). Therefore, targeting the cell cycle machinery is an attractive anticancer strategy. Previous studies have shown that OSW-1 significantly suppresses the proliferation of various cancer cells (Zhan et al., 2021). Jin et al. speculated that OSW-1 could greatly affect the expression of cell cycle-related genes after examining the changes in gene expression of hepatocellular carcinoma cells incubated with OSW-1 *in vitro* (Jin et al., 2013). In 2019, Iguchi et al. found that the cell cycle of leukemia HL-60 cells treated with OSW-1 was arrested at the G2/M phase (Iguchi et al., 2019). In our study, we found that OSW-1 inhibited the viability and proliferation of glioma cells and arrested cell cycle at G2/M by downregulating the expression of cycling B1 and CDK1 and upregulating the expression of P21. Cell cycle regulation is a complex regulatory network involving changes in a series of cell cycle-related proteins (Strzyz, 2016). Different cyclins can combine with their corresponding cyclin-dependent kinases (CDK) to form complexes with kinase activity, thus promoting the progression of the cell cycle. The cyclin B1-CDK1 complex works at the end of the G2 phase, helping cells to cross the G2/M checkpoint and enter the M phase (Matthews et al., 2022). P21, as a cyclin-dependent kinase inhibitor (CKI), can negatively regulate the cell cycle by inactivating and maintaining the inactive state of mitotic cyclin-CDK complexes, contributing to permanent G2 arrest (Charrier-Savournin et al., 2004).

Apoptosis is a form of programmed cell death that usually acts as a homeostatic mechanism to maintain cell populations in tissues (Elmore, 2007). Once out of control, it occurs commonly in cancers, and cells would gain immortality and the ability to proliferate without death threats (Wong, 2011). Apoptosis can be divided into two main pathways: intrinsic (mitochondria-mediated) and extrinsic (death receptor-mediated). Both pathways involve the activation of a series of caspase reactions, such as caspase-3/8/9. Parp-1 is cleaved by caspase-3 during apoptosis, which promotes cell disintegration and can serve as a marker of apoptosis (Oliver et al., 1998). In our study, OSW-1 was capable of significantly increasing the apoptotic cell ratio of glioma cells, and discovered that the expression level of cleaved caspase-3/9 and cleaved PARP-1 increased in a dose-dependent manner using a western blot assay. The results were consistent with the findings of previous studies on OSW-1 in other cancer cells, although the specific mechanisms were not exactly the same (Zhan et al., 2021). In colon cancer cells, OSW-1 induces apoptosis via the classical intrinsic pathway (Zhang et al., 2017). However, Iguchi et al. found that OSW-1 induces apoptosis in leukemia cells via a mitochondria-independent pathway (Iguchi et al., 2019). Furthermore, apoptosis via a caspase-8-dependent pathway, which is regarded as a classical extrinsic pathway, was observed in Chinese hamster ovary cells by Zhu et al. (Zhu et al., 2005). Notably, OSW-1 can also induce apoptosis via a Golgi stress-induced mechanism in cervical cancer cells (Kimura et al., 2019). The reason for this phenomenon may be that the different cell lines are uniquely affected by OSW-1.

In recent years, it has been shown that the alterations in PI3K/AKT signaling pathway components are frequent in human cancers (Fresno Vara et al., 2004) and account for approximately 88% of patients with GBM (Cancer Genome Atlas Research Network, 2008). Aberrant activation of this pathway is closely related to the occurrence, proliferation, growth, apoptosis, invasion, metastasis, epithelial-mesenchymal transformation, stem cell-like phenotype, immune microenvironment, and drug resistance of cancer cells (Jiang et al., 2020). Given that this pathway is mutated in the majority of GBM and that the hyper-activated AKT helps glioma cells grow uncontrollably, evade apoptosis, and enhance tumor invasion, targeting this pathway has been an attractive therapeutic strategy for glioma (McDowell et al., 2011; Zhang et al., 2021). In this study, network pharmacology was used to predict the potential anti-glioma mechanisms of OSW-1. We found that the PI3K/AKT pathway was the most significant pathway, with 38 enriched intersecting genes and the hub genes PIK3R1 and AKT1. Therefore, we speculated that the anti-glioma mechanisms of OSW-1 might be associated with inactivation of the PI3K/AKT signaling pathway by decreasing the relative levels of phosphorylated PI3K and AKT1. To verify this hypothesis, western blotting was performed to detect the expression levels of PI3K, p-PI3K, AKT1, and p-AKT1. These results are consistent with our finding that OSW-1 significantly inhibits the phosphorylation of PI3K and AKT1. However, the PI3K activator 740Y-P reversed the inactivation of the PI3K/AKT signaling pathway caused by OSW-1.

To further confirm the anti-glioma effect of OSW-1 *in vivo*, we established a xenograft tumor model of nude mice by transplanting LN18 cells. The results confirmed the suppressive effect of OSW-1 on the transplanted tumor with limited side effects on the weight of nude mice. Likewise, OSW-1 had a significant inhibitory effect on the activation of PI3K/AKT signaling pathway *in vivo*. Our findings indicate that OSW-1 may be used as a novel inhibitor of the PI3K/AKT pathway in experimental studies and clinical applications. However, the pharmacokinetics of OSW-1 is a blank of current research. Since OSW-1 remains many issues, such as the drug movement within the body, including the time course of absorption, distribution, metabolism and excretion (ADME), bioavailability, plasma concentration, and blood-brain barrier permeability, to be solved, its way to becoming a true clinical anti-glioma drug is still far. It is the limitation of our study that we didn’t perform more vigorous pharmacology experiments to uncover its pharmacokinetic parameters *in vivo* and drug-like properties of OWS-1. In future, more *in vivo* experiments are needed to refine the pharmacokinetics and pharmacology of OSW-1, which require the joint efforts of researchers from different professions, so that it can further move to clinical application (Zhan et al., 2021).

## Conclusion

In this study, we investigated the anticancer effects of OSW-1 on glioma cells *in vitro* and *in vivo* and predicted its potential mechanisms using a network pharmacology approach. We found that OSW-1 inhibited cell viability and proliferation, induced cell apoptosis, and arrested the cell cycle at the G2/M phase and that OSW-1-induced inhibition of the PI3K/AKT signaling pathway plays a crucial role in these anticancer effects. These findings provide evidence that OSW-1 is a potential therapeutic agent for glioma and warrants further study. However, OSW-1 is still far from becoming a real anti-glioma agent for some unsolved issues about pharmacokinetics and pharmacology. This will require the joint efforts of researchers from different professions, worldwide.

## Data Availability

The original contributions presented in the study are included in the article/supplementary material, further inquiries can be directed to the corresponding authors.
