# Correction: Anticancer effects of OSW-1 on glioma cells *via* regulation of the PI3K/AKT signal pathway: a network pharmacology approach and experimental validation *in vitro* and *in vivo*

**DOI:** 10.3389/fphar.2025.1754450

**Published:** 2025-12-11

**Authors:** Zhixin Zhan, Ziqiang Liu, Chaochao Zhang, Haijun Gao, Jiacheng Lai, Yong Chen, Haiyan Huang

**Affiliations:** Department of Neurosurgery, The First Hospital of Jilin University, Changchun, China

**Keywords:** OSW-1, glioma, network pharmacology, cell cycle, apoptosis, Pi3k/ AKt

There was a mistake in Figure 8E as published. The Total AKT1 band was inadvertently replaced with a duplicated β-actin band during figure assembly. The corrected Figure 8 appears below.

**FIGURE 8:**
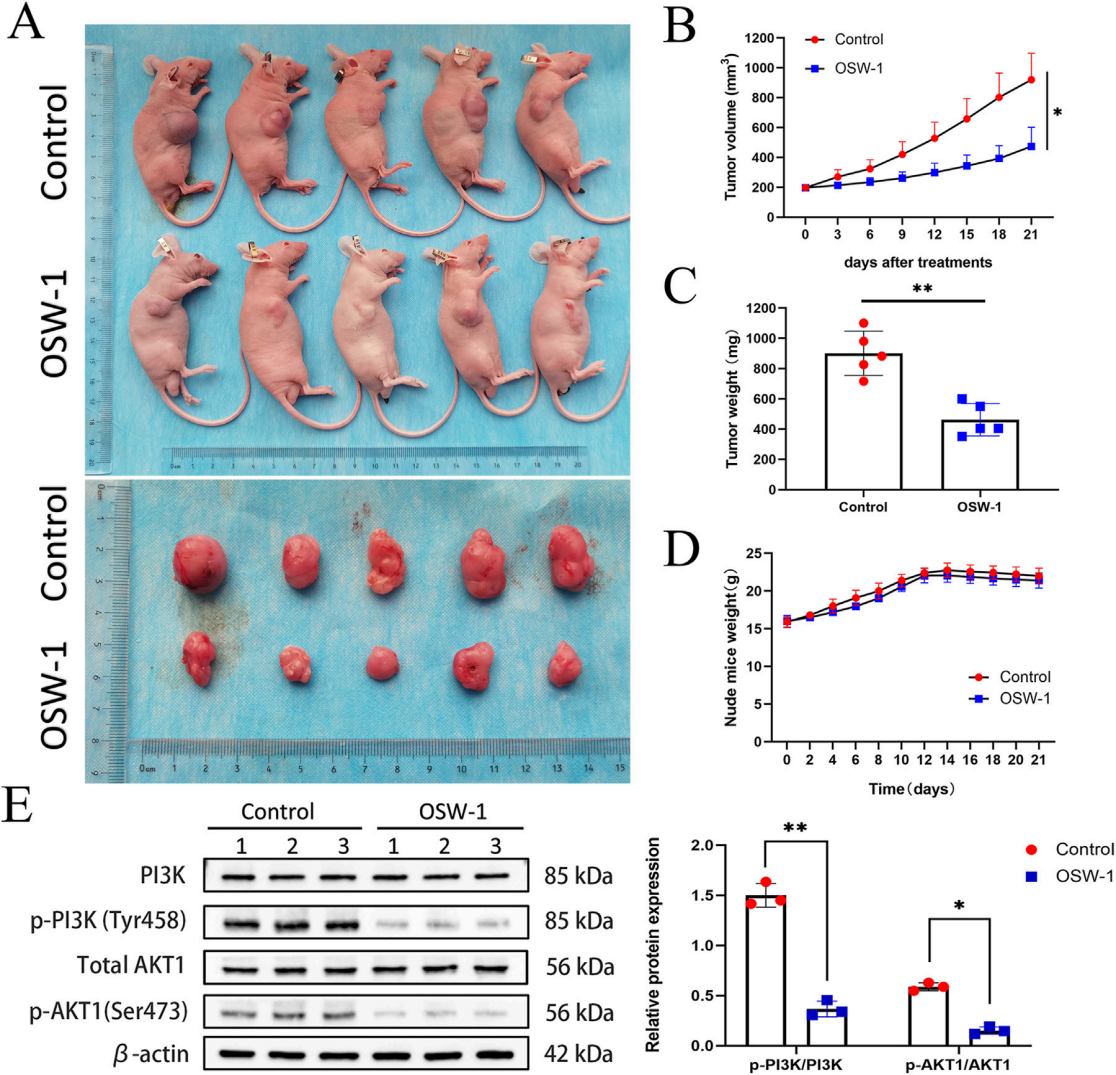
OSW-1 inhibited tumor growth in the xenograft model mice **(A)** The tumors of control mice and OSW-1-treated mice were extracted and photographed **(B,C)** Inhibitions in the average volume and weight of glioma xenografts were observed after 21 days of OSW-1 treatment **(D)** Body weight changes of mice during 21 days of exposure **(E)** Western blotting of tumor tissue lysates. *p < 0.05, **p < 0.01, compared to the control group.

The original article has been updated.

